# Supplementary material for: Resolving biology’s dark matter: species richness, spatiotemporal distribution, and community composition of a dark taxon
Source: BMC Biol. 2024 Sep 27;22:215. doi: 10.1186/s12915-024-02010-z (PMC11438253; doi:10.1186/s12915-024-02010-z)
Supplement: Supplementary file 1 — Supplementary Material 1: Additional file 1: Figures S1–S13, Tables S1–S6. Fig. S1 Map of study sites. Fig. S2 Timeline of analysed samples. Fig. S3 Chao1 estimates of scuttle fly species richness per zone. Fig. S4 Species accumulation curve of scuttle flies by season. Fig. S5 Consistency in community patterning, as based on different clustering thresholds for species delimitation. Fig. S6 NMDS plot of all samples (species, threshold of 100 specimens) with samples colour-coded according to time period. Fig. S7 Violin plots showing potential scale reduction factors (PSRF) for the beta and omega parameters of four HMSC models: species occurrence (1), species abundance (2), haplotype occurrence (3), and haplotype abundance (4). Fig. S8 Variance explained by fixed and random effects in HMSC models of the presence/absence or abundance of either haplotypes or species proxies. Fig. S9 Predicted mean prevalence and log(abundance) of species proxies as a function of forest or woodland cover (%) and mean annual temperature (°C) during late summer in HMSC models. Fig. S10 Pairwise residual associations among haplotypes and species proxies in space and time as detected in HMSC models. Fig. S11 Pairwise compositional turnover as a function of intervening geographic distance for each time period in the species and haplotype datasets. Fig. S12 Nestedness values scored along geographic distance gradients for each time period among the species and haplotype datasets. Fig. S13 Total beta-diversity values scored along geographic distance gradients for each time period among the species and haplotype datasets. Table S1 Sample information. Table S2 Distinctness of scuttle fly communities across zones, as based on different clustering thresholds for species delimitation. Table S3 Similarity of scuttle fly communities across zones, as based on different clustering thresholds for species delimitation. Table S4 Distinctness of scuttle fly communities across time periods. Table S5 Similarity [file 12915_2024_2010_MOESM1_ESM.pdf]

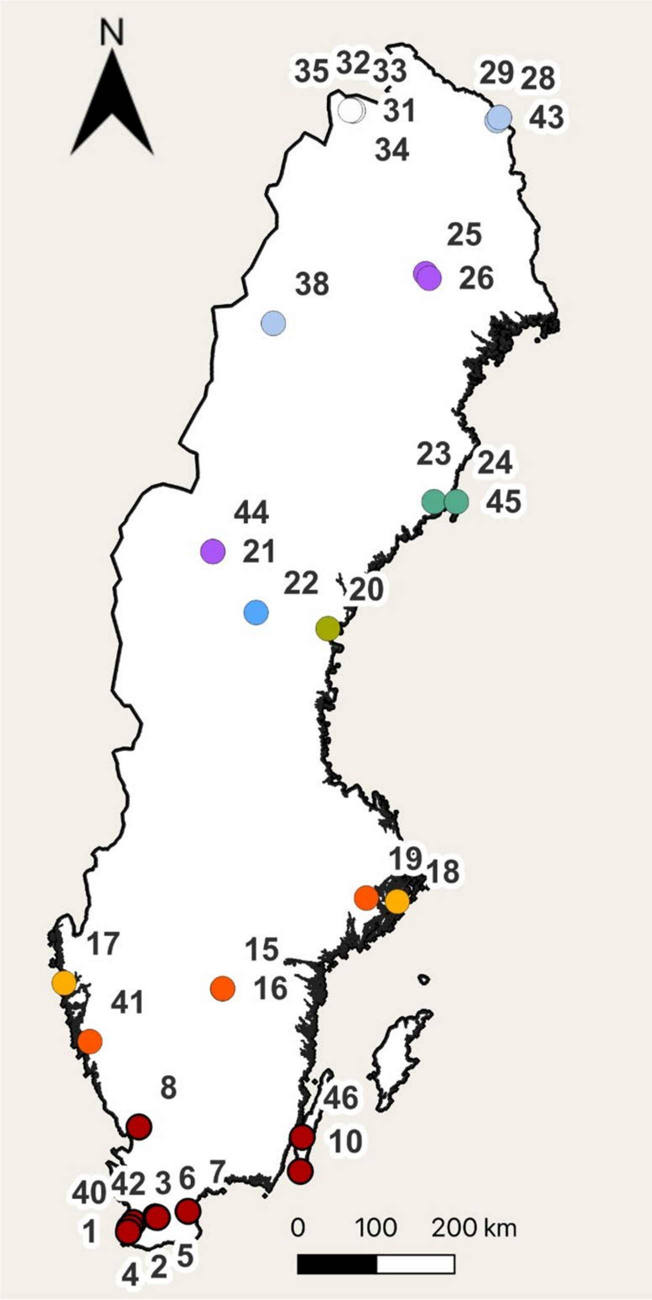

Supplementary Figure S1. Map of study sites.

A map of the 37 study sites at which scuttle flies were sampled, colour coded according to Swedish horticultural zones (see Fig. 1).

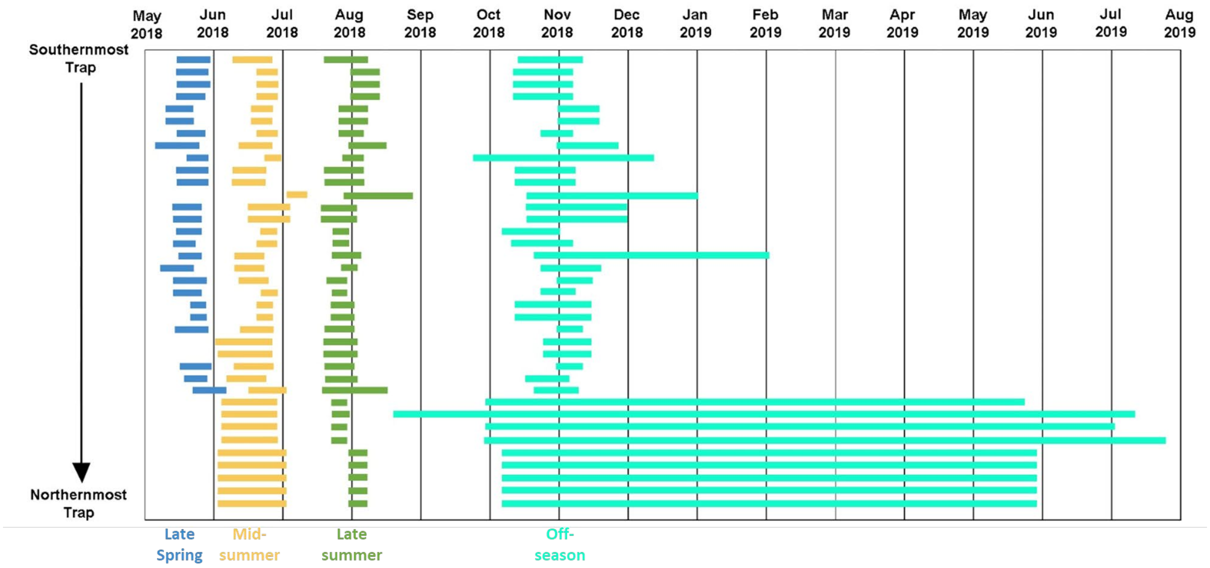

Supplementary Figure S2. Timeline of analysed samples.

Four sequential samples from each site were selected to represent scuttle fly communities in the late spring (mid-late May, in blue), midsummer (June, in yellow), late summer (late July – early August, in green) and the offseason (last sample of the year, in teal). Many of the traps in the north are missing late spring samples as they were not set up until later in the season. These same sites have long offseason samples as they were not collected until the following spring.

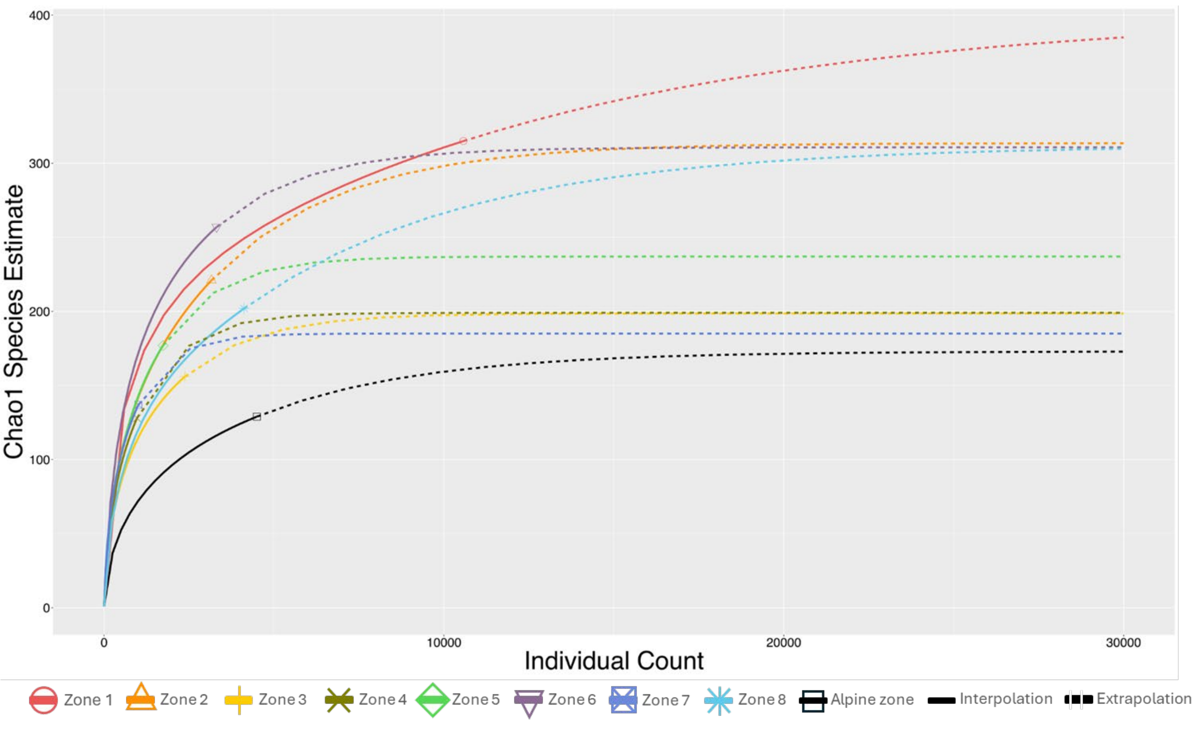

Supplementary Figure S3. Chao1 estimates of scuttle fly species richness per zone.

Colour-coding is according to plant hardiness zones; for a map of the zones using the same colour codes, see Fig. 1.

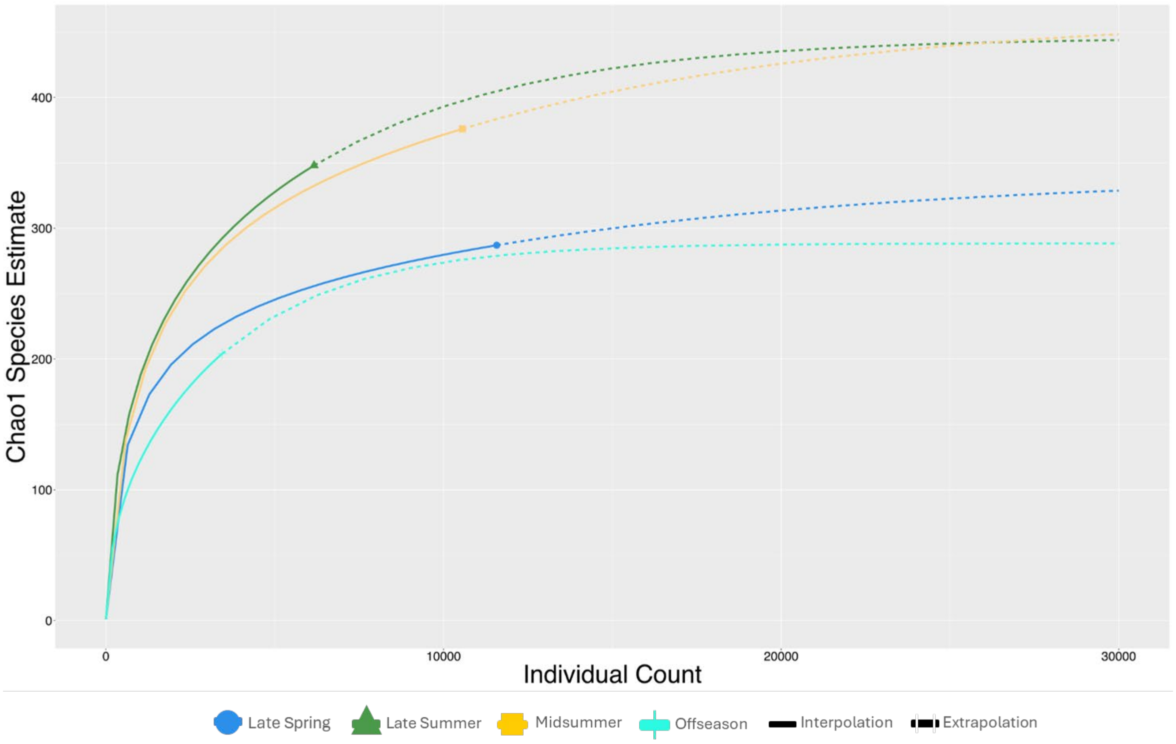

Supplementary Figure S4. Species accumulation curves of scuttle flies by season.

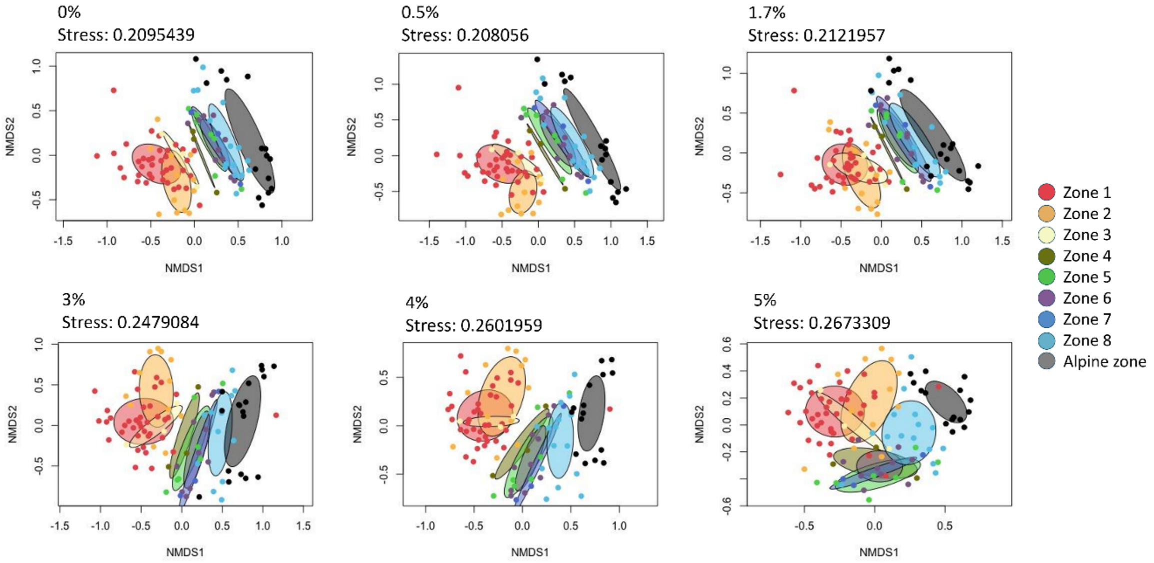

Supplementary Figure S5. Consistency in community patterning, as based on different clustering threshold for species delimitation.

Shown are NMDS plots with scuttle fly mOTUs calculated at different thresholds with ellipses coloured according to plant hardiness zones. The pattern is consistent from 0-1.7%, after which there is a clear shift and as the threshold increase, the pattern begins to blur as species are lumped. Colour-coding reflects plant hardiness zones, shown in the same colours in Fig. 1.

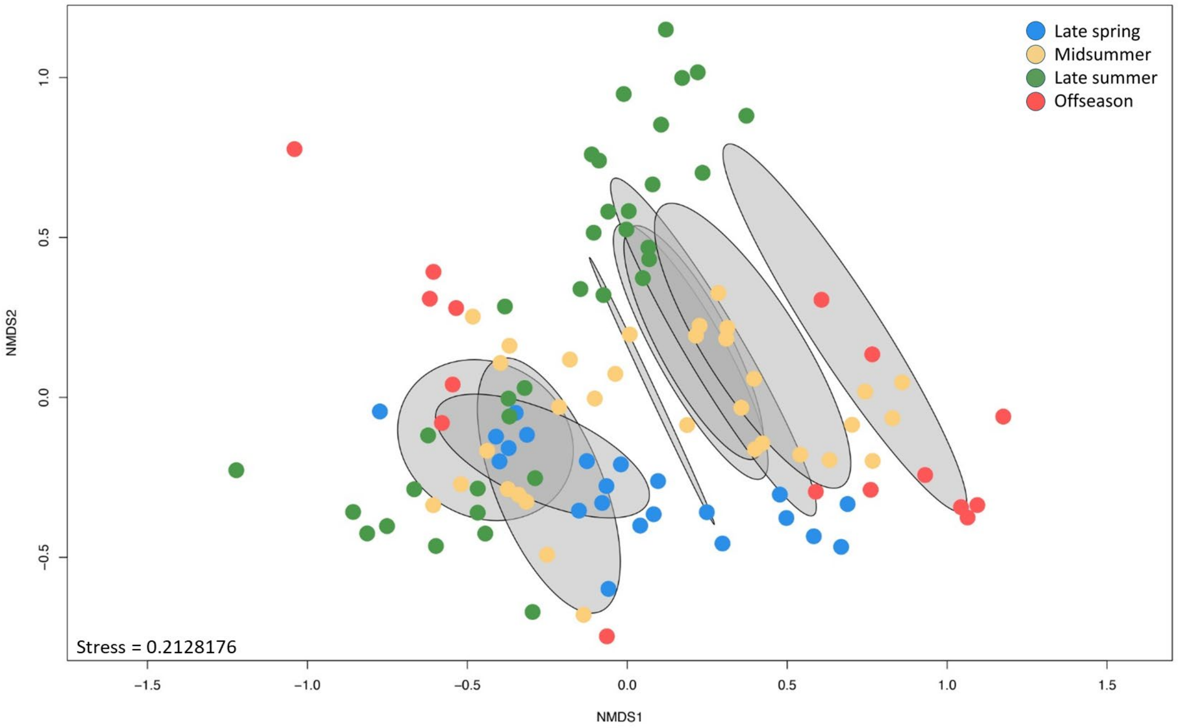

Supplementary Figure S6. NMDS plot of all samples (species, threshold of 100 specimens) with samples colour-coded according to time-period.

Except for the “Offseason” period, seasonal catches run linearly through zonal ellipses, in grey.

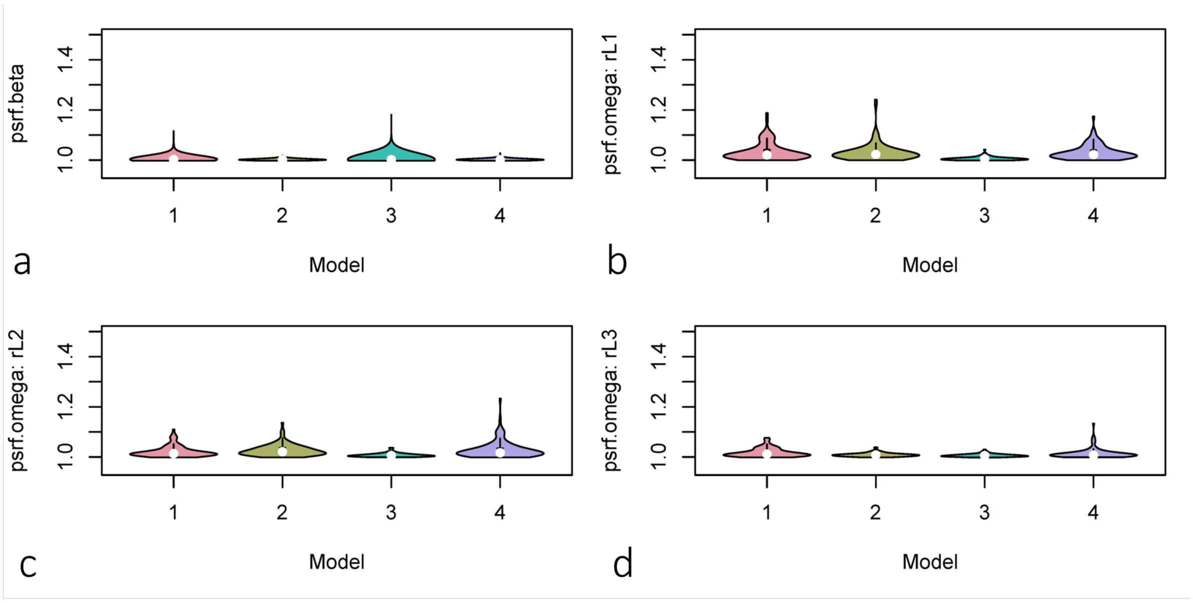

Supplementary Figure S7. Violin plots showing potential scale reduction factors (PSRF) for the beta and omega parameters of four HMSC models: species occurrence (1), species abundance (2), haplotype occurrence (3), and haplotype abundance (4).

Shown are the PSRF values for (a) fixed effect (β) parameters, (b) random effect (Ω) parameter space, (c) random effect (Ω) ***parameter*** *year and (d) random effect (Ω) parameter sample-level.*

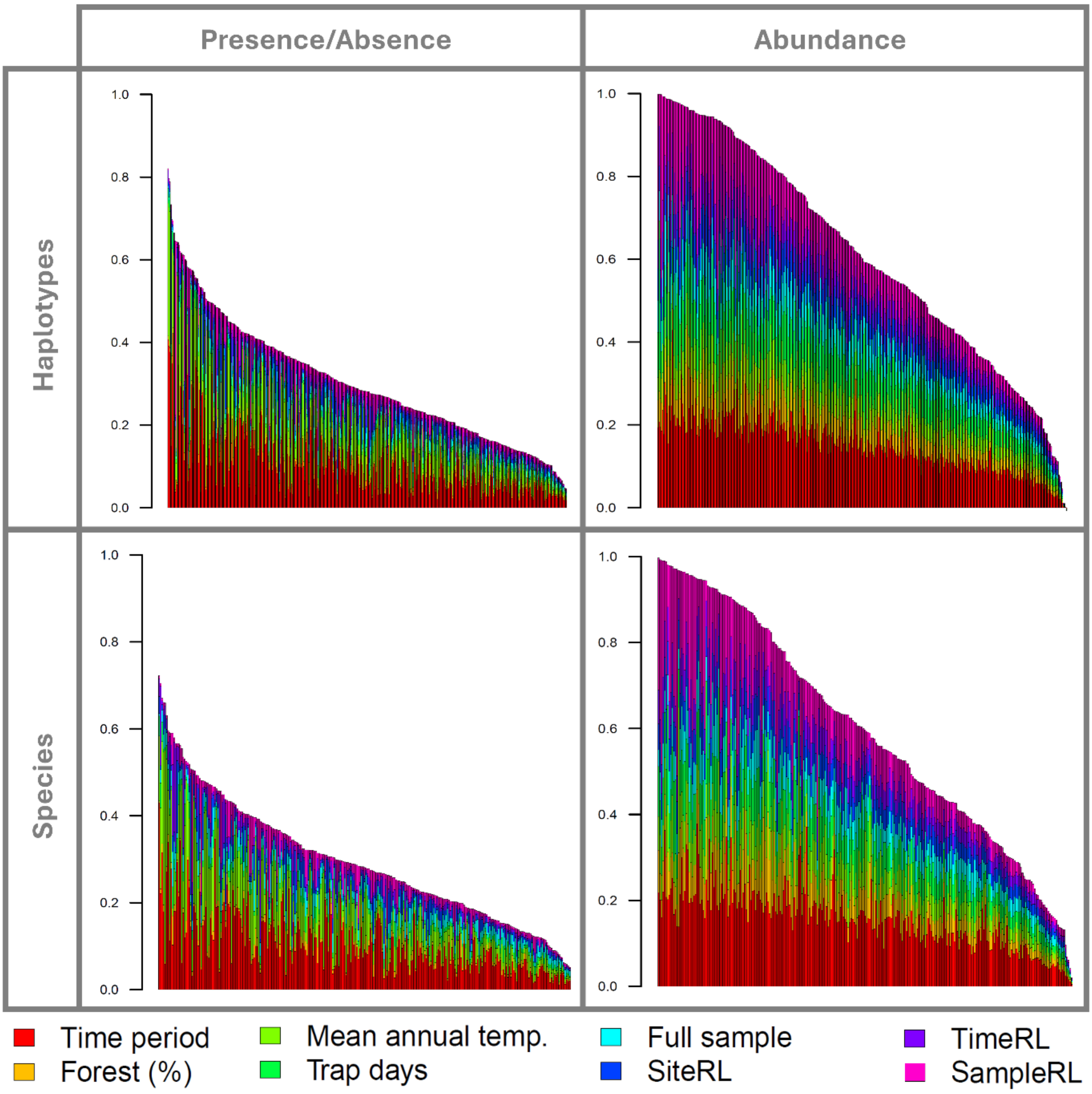

Supplementary Figure S8. Variance explained by fixed and random effects in HMSC models of the presence/absence or abundance of either haplotypes or species proxies.

Each bar represents the variance partitioning result for a single haplotype or mOTU, illustrated in descending order of total explained variance (%).

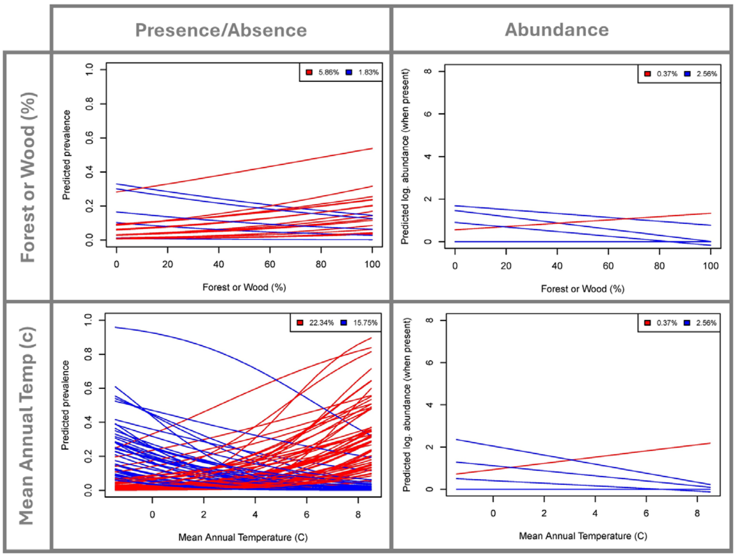

Supplementary Figure S9. Predicted mean prevalence and log(abundance) of species proxies as a function of forest or woodland cover (%) and mean annual temperature (°C) during late summer in HMSC models.

Only those mOTUs are illustrated for which a directional trend was predicted with > 95% posterior support. The numbers in the legend represent the percentages of mOTUs, of the total modelled, that were predicted to have a positive (red) or negative (blue) response to the focal covariate. These are marginal predictions, where values for all covariates apart from the focal covariate and sampling season (late summer) were fixed at their mean value in the dataset. The results were similar for other sampling seasons as well, but only those for late summer are illustrated.

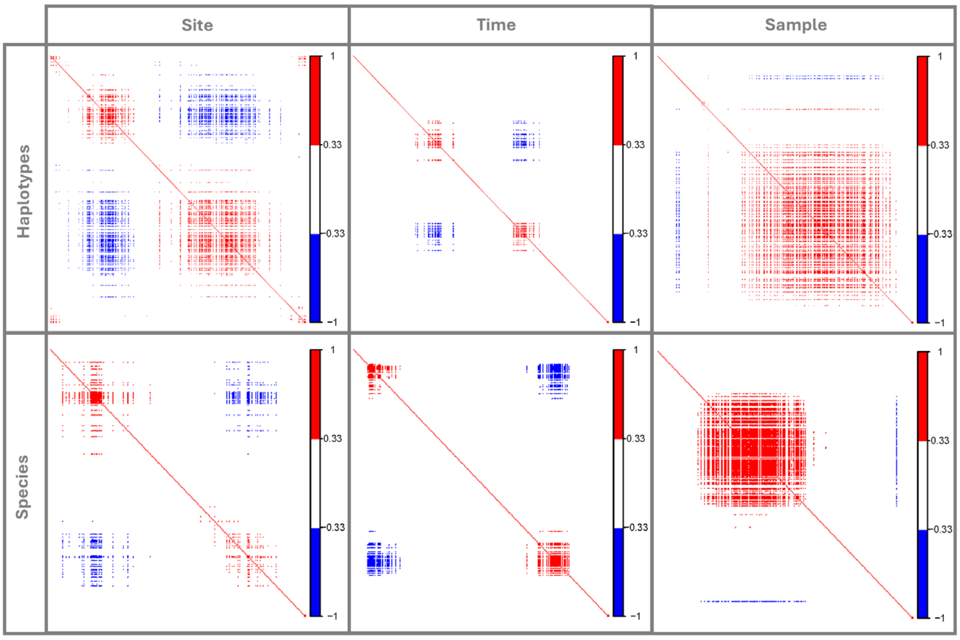

Supplementary Figure S10. Pairwise residual associations among haplotypes and species proxies in space and time as detected in HMSC models.

Taxa are ordered on the x- and y-axes such that positive and negative residual associations are in blocks so that their prevalence across the datasets is easy to compare.

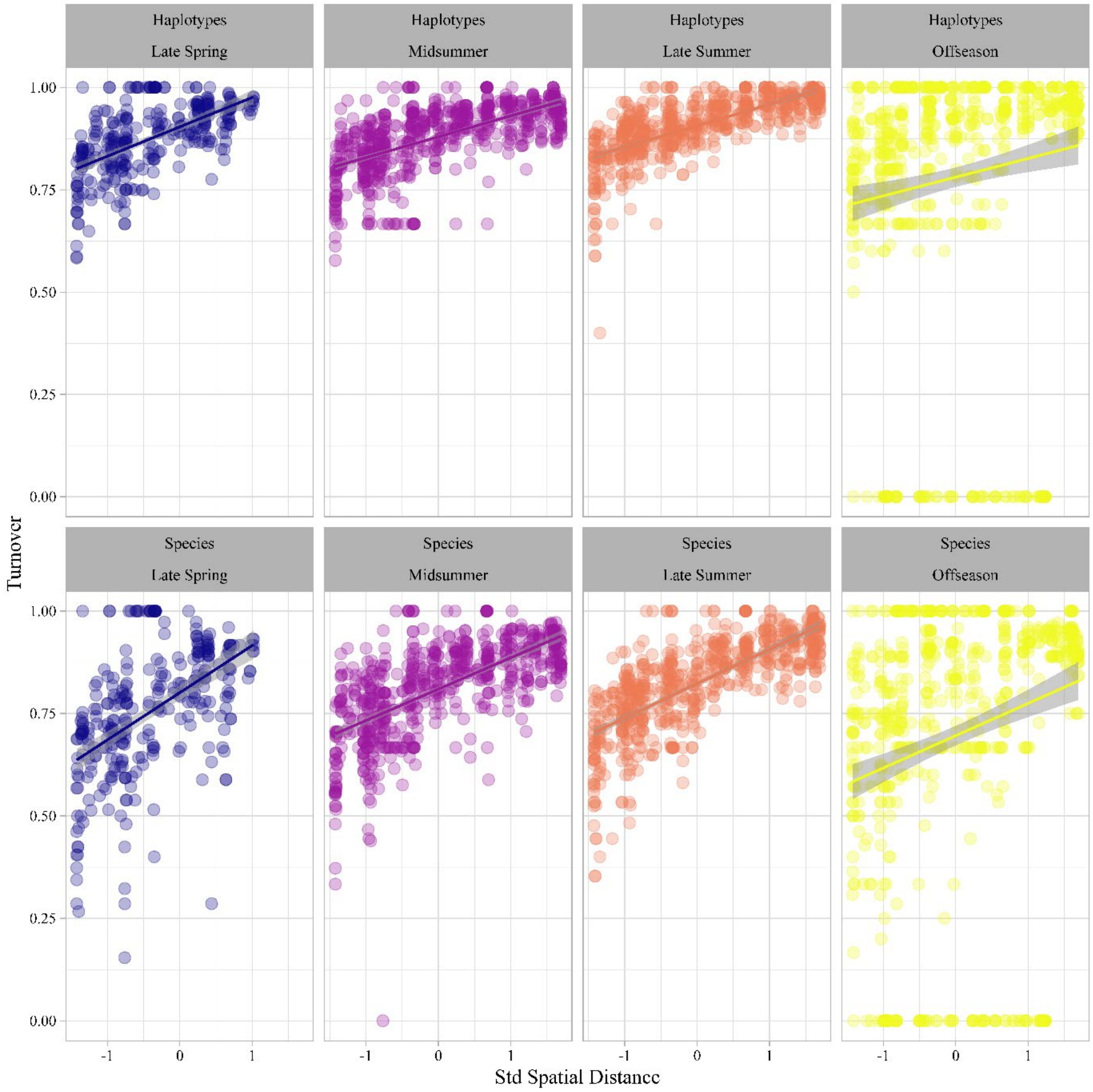

Supplementary Figure S11. Pairwise compositional turnover as a function of intervening geographic distance for each time period in the mOTU and Haplotype datasets.

Following Baselga (2010), we partitioned overall Jaccard dissimilarity between samples from each time period (Late spring, Midsummer, Late summer, Offseason) into its turnover and nestedness components using either species (1.7% mOTUs) or haplotypes. Shown in each panel here are scatterplots of turnover of haplotypes (upper panels) vs mOTUs (lower panels) per time period between each pair of sampling sites as a function of their standardized intervening geographical distance.

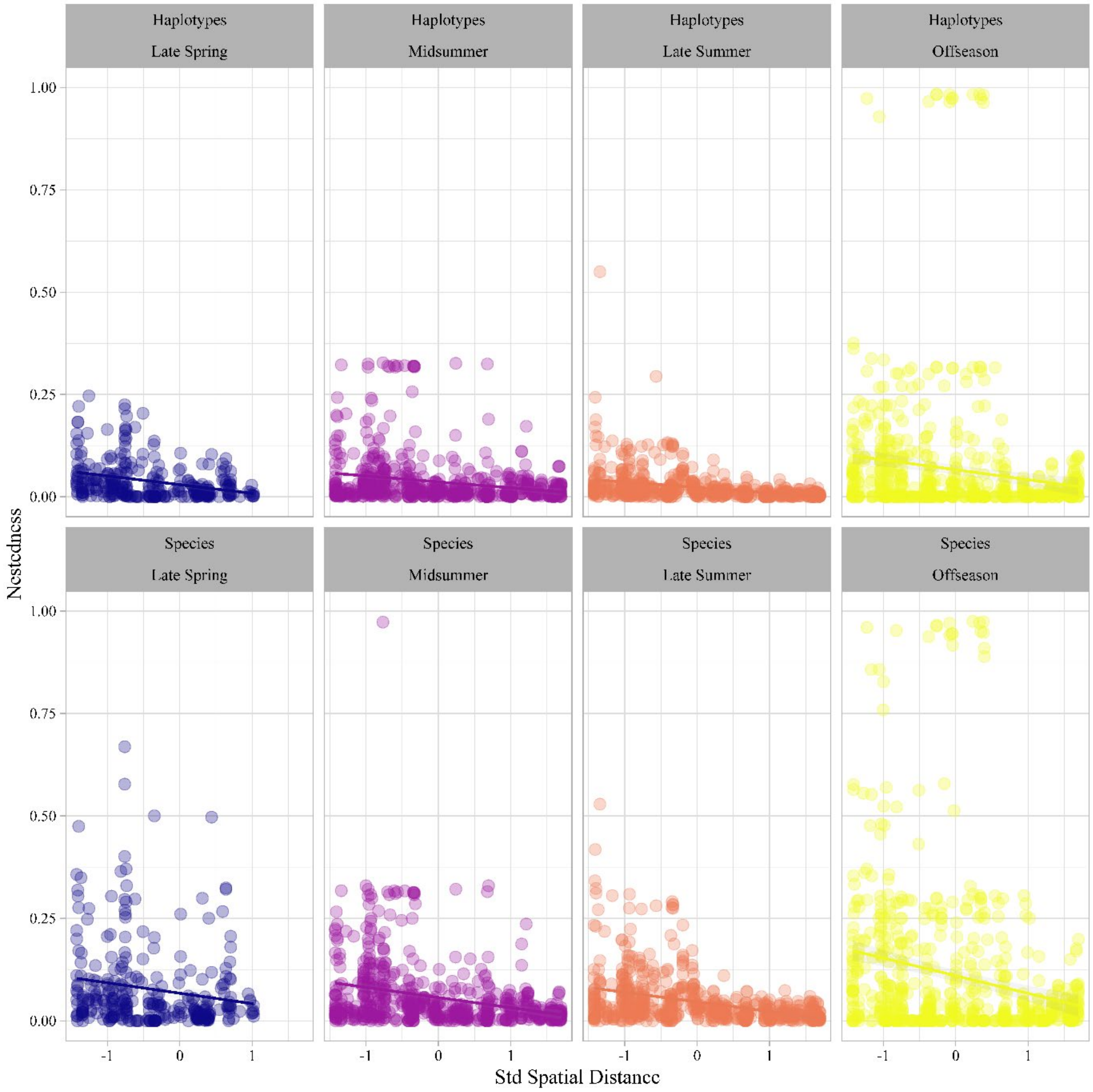

Supplementary Figure S12. Nestedness values scored along geographic distance gradients for each time period among the Species and Haplotype datasets.

Following Baselga (2010), we partition overall Jaccard dissimilarity into its turnover and nestedness components using either species (1.7% mOTUs) or haplotypes recorded at each different time period. Shown in each panel are the scatterplots of nestedness between each pair of sampling sites (for each dataset) at each time period, as a function of their standardized intervening geographical distance.

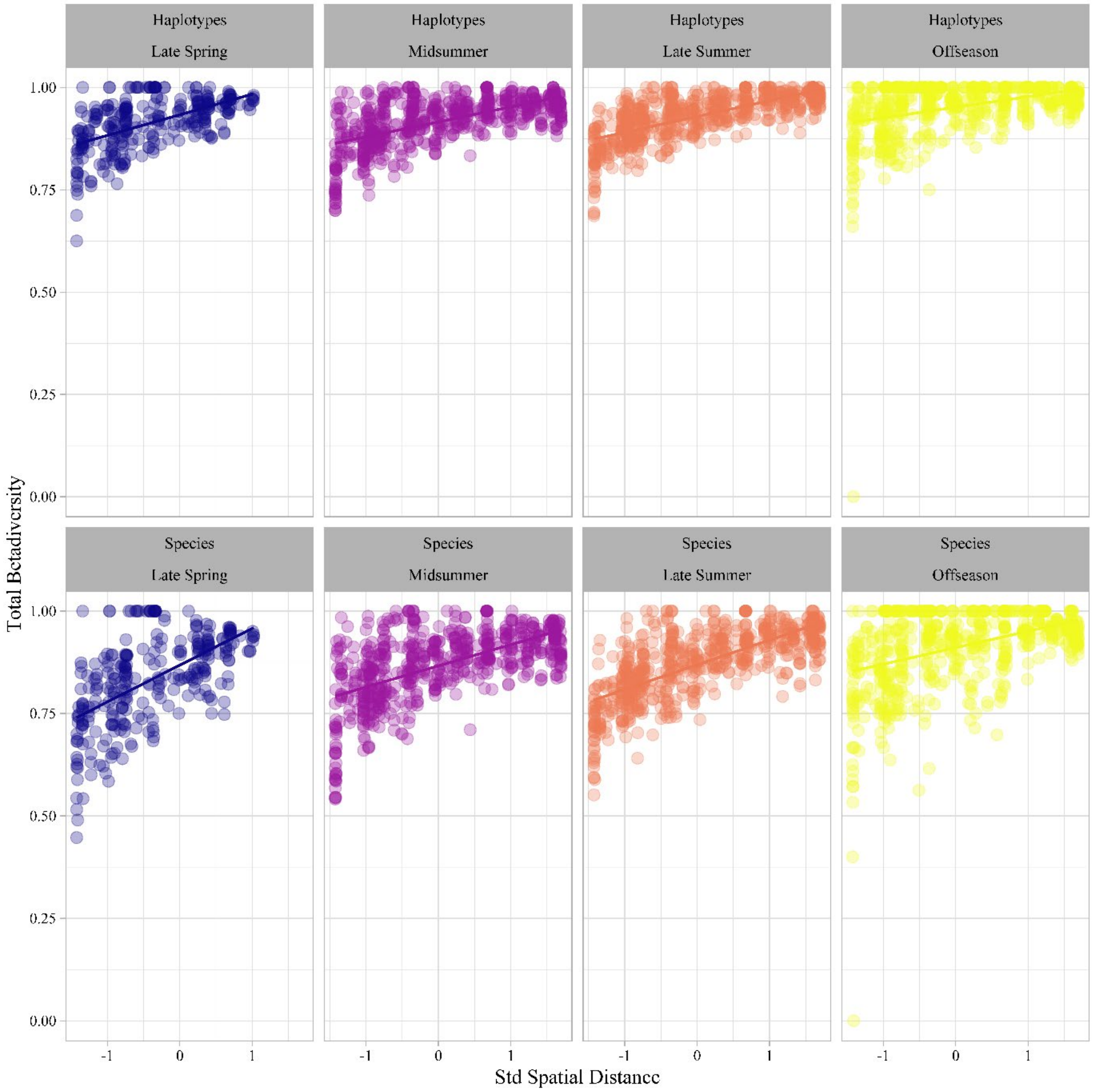

Supplementary Figure S13. Total beta-diversity values scored along geographic distance gradients for each time period among the Species and Haplotype datasets.

Shown in each panel are the scatterplots of pairwise overall community dissimilarity between each pair of sampling sites (for each dataset) at each time period, against their spatial separation (standardized Euclidean distance between sample site coordinates). To illustrate the (lack of) impact of species delimitation criteria used, we show the same patterns using haplotypes (upper panels) vs species (1.7% mOTUs, lower panels).

Supplementary Table S1. Sample information.

In this table, we provide metadata on each sample, grouped into the following columns: Site ID: the identity of the site; Start date: the first day of the sampling period (format day/month/year); End date: the first day of the sampling period (format day/month/year); Trapping days: length of sampling period (i.e. end date minus starting date); Latitude (in decimal degrees); Longitude (in decimal degrees); County (i.e., region within Sweden); Barcodes: the number of barcode sequences successfully retrieved; Sample sequence: information on whether all individuals in the sample were sequenced (Y) or not (N; see Supplementary Text S2); Time period: phase of summer phenology, grouped into: Late spring (May), midsummer (June), late summer (July/August), and offseason (the last sample of the season, collected in November or December 2018 or spring of 2019)(time periods are approximate, consult table for exact sample dates); Horticultural zone: plant hardiness zones defined by the Swedish Horticultural Society (Riksförbundet Svensk Trädgård 2018), with colours corresponding to those used in Fig. 1 of the main text); Avg annual temperature: mean annual temperature of the climate zone, derived from the Swedish Meteorological and Hydrological Institute (SMHI; smhi.se) for time period 1991-2020; Avg annual precipitation: mean annual precipitation of the climate zone, derived from the Swedish Meteorological and Hydrological Institute (SMHI; smhi.se) for time period 1991-2020.

Site ID	Sample ID	Start date	End date	Trapping Days	Latitude	Longitude	County	Barcodes	Entire sample sequenced	Time period	Horticultural zone	Avg. annual temperature	Avg. annual precipitation
1	M01013	12.10.2018	10.11.2018	29	55,52527	12,913264	Skåne	61	Y	Offseason	I (red)	8-10	500-600
1	M01025	14.05.2018	28.05.2018	14	55,52527	12,913263	Skåne	312	Y	Late Spring	I (red)	8-10	500-600
1	M01030	10.06.2018	25.06.2018	15	55,52527	12,913263	Skåne	214	N	Midsummer	I (red)	8-10	500-600
1	M01035	19.07.2018	07.08.2018	19	55,52527	12,913263	Skåne	168	N	Late Summer	I (red)	8-10	500-600
2	M02006	15.05.2018	25.05.2018	10	55,56939	12,926716	Skåne	535	Y	Late Spring	I (red)	8-10	500-600
2	M02010	20.06.2018	28.06.2018	8	55,56939	12,926716	Skåne	184	N	Midsummer	I (red)	8-10	500-600
2	M02014	30.07.2018	13.08.2018	14	55,56939	12,926716	Skåne	155	N	Late Summer	I (red)	8-10	500-600
2	M02020	10.10.2018	05.11.2018	26	55,56939	12,926717	Skåne	35	Y	Offseason	I (red)	8-10	500-600
3	M03006	15.05.2018	27.05.2018	12	55,63332	13,009706	Skåne	179	Y	Late Spring	I (red)	8-10	500-600
3	M03010	20.06.2018	28.06.2018	8	55,63332	13,009706	Skåne	18	Y	Midsummer	I (red)	8-10	500-600
3	M03014	30.07.2018	13.08.2018	14	55,63332	13,009706	Skåne	175	N	Late Summer	I (red)	8-10	500-600
3	M03019	10.10.2018	05.11.2018	26	55,63332	13,009707	Skåne	111	Y	Offseason	I (red)	8-10	500-600
4	M04006	15.05.2018	25.05.2018	10	55,57838	12,948558	Skåne	767	N	Late Spring	I (red)	8-10	500-600
4	M04010	20.06.2018	28.06.2018	8	55,57838	12,948558	Skåne	261	N	Midsummer	I (red)	8-10	500-600
4	M04014	30.07.2018	13.08.2018	14	55,57838	12,948558	Skåne	165	N	Late Summer	I (red)	8-10	500-600
4	M04020	10.10.2018	05.11.2018	26	55,57838	12,948559	Skåne	182	N	Offseason	I (red)	8-10	500-600
5	M05006	10.05.2018	19.05.2018	9	55,70427	13,452154	Skåne	525	N	Late Spring	I (red)	8-10	600-700
5	M05012	16.06.2018	25.06.2018	9	55,70427	13,452154	Skåne	168	N	Midsummer	I (red)	8-10	600-700
5	M05019	27.07.2018	07.08.2018	11	55,70427	13,452154	Skåne	172	N	Late Summer	I (red)	8-10	600-700
5	M05029	30.10.2018	18.11.2018	19	55,70427	13,452155	Skåne	86	Y	Offseason	I (red)	8-10	600-700
6	M06005	12.05.2018	19.05.2018	7	55,69799	13,496168	Skåne	421	Y	Late Spring	I (red)	8-10	600-700
6	M06009	16.06.2018	25.06.2018	9	55,69799	13,496168	Skåne	178	N	Midsummer	I (red)	8-10	600-700
6	M06016	27.07.2018	07.08.2018	11	55,69799	13,496168	Skåne	178	N	Late Summer	I (red)	8-10	600-700
6	M06026	30.10.2018	18.11.2018	19	55,69799	13,496169	Skåne	24	Y	Offseason	I (red)	8-10	600-700
7	M07004	14.05.2018	23.05.2018	9	55,77455	14,121564	Skåne	556	N	Late Spring	I (red)	8-10	700-800
7	M07008	19.06.2018	28.06.2018	9	55,77455	14,121564	Skåne	181	N	Midsummer	I (red)	8-10	700-800
7	M07012	26.07.2018	04.08.2018	9	55,77455	14,121564	Skåne	179	N	Late Summer	I (red)	8-10	700-800
7	M07020	21.10.2018	06.11.2018	16	55,77455	14,121565	Skåne	182	N	Offseason	I (red)	8-10	700-800
8	M08002	07.05.2018	21.05.2018	14	56,73076	13,067036	Halland	195	Y	Late Spring	I (red)	6-8	1100-1200
8	M08005	12.06.2018	27.06.2018	15	56,73076	13,067036	Halland	161	N	Midsummer	I (red)	6-8	1100-1200
8	M08009	29.07.2018	15.08.2018	17	56,73076	13,067036	Halland	115	N	Late Summer	I (red)	6-8	1100-1200
8	M08013	29.10.2018	27.11.2018	29	56,73077	13,067037	Halland	9	Y	Offseason	I (red)	6-8	1100-1200
10	M10001	18.05.2018	25.05.2018	7	56,22829	16,441877	Kalmar	482	Y	Late Spring	I (red)	8-10	400-500
10	M10005	20.06.2018	29.06.2018	9	56,22829	16,441877	Kalmar	170	N	Midsummer	I (red)	8-10	400-500
10	M10010	27.07.2018	03.08.2018	7	56,22829	16,441877	Kalmar	185	N	Late Summer	I (red)	8-10	400-500
10	M10017	26.09.2018	21.12.2018	86	56,22829	16,441877	Kalmar	187	N	Offseason	I (red)	8-10	400-500
15	M15002	15.06.2018	03.07.2018	18	58,33087	14,817465	Östergötland	313	N	Midsummer	II (orange)	6-8	500-600
15	M15004	18.07.2018	06.08.2018	19	58,33087	14,817465	Östergötland	165	N	Late Summer	II (orange)	6-8	500-600
15	M15010	14.10.2018	30.11.2018	47	58,33087	14,817466	Östergötland	134	Y	Offseason	II (orange)	6-8	500-600
15	M15026	14.05.2018	24.05.2018	10	58,33087	14,817465	Östergötland	516	N	Late Spring	II (orange)	6-8	500-600
16	M16002	14.05.2018	24.05.2018	10	58,33338	14,82316	Östergötland	547	N	Late Spring	II (orange)	6-8	500-600
16	M16004	15.06.2018	03.07.2018	18	58,33338	14,82316	Östergötland	188	N	Midsummer	II (orange)	6-8	500-600
16	M16008	18.07.2018	06.08.2018	19	58,33338	14,82316	Östergötland	156	N	Late Summer	II (orange)	6-8	500-600
16	M16014	16.10.2018	30.11.2018	45	58,33338	14,82317	Östergötland	174	N	Offseason	II (orange)	6-8	500-600
17	M17005	17.05.2018	23.05.2018	6	58,3442	11,334593	Västra Götaland	50	Y	Late Spring	III (gold)	8-10	800-900
17	M17008	11.06.2018	22.06.2018	11	58,3442	11,334593	Västra Götaland	27	Y	Midsummer	III (gold)	8-10	800-900
17	M17012	25.07.2018	09.08.2018	15	58,3442	11,334593	Västra Götaland	48	Y	Late Summer	III (gold)	8-10	800-900
17	M17018	19.10.2018	05.02.2019	109	58,3442	11,334594	Västra Götaland	10	Y	Offseason	III (gold)	8-10	800-900
18	M18005	13.05.2018	22.05.2018	9	59,27815	18,761432	Stockholm	1815	Y	Late Spring	III (gold)	6-8	500-600
18	M18009	11.06.2018	22.06.2018	11	59,27815	18,761432	Stockholm	180	N	Midsummer	III (gold)	6-8	500-600
18	M18015	29.07.2018	06.08.2018	8	59,27815	18,761432	Stockholm	176	N	Late Summer	III (gold)	6-8	500-600
18	M18022	21.10.2018	16.11.2018	26	59,27815	18,761433	Stockholm	69	Y	Offseason	III (gold)	6-8	500-600
19	M19007	17.05.2018	24.05.2018	7	59,33624	18,067797	Stockholm	1	Y	Late Spring	II (orange)	6-8	500-600
19	M19012	21.06.2018	28.06.2018	7	59,33624	18,067797	Stockholm	2	Y	Midsummer	II (orange)	6-8	500-600
19	M19017	25.07.2018	02.08.2018	8	59,33624	18,067797	Stockholm	11	Y	Late Summer	II (orange)	6-8	500-600
19	M19027	05.10.2018	01.11.2018	27	59,33624	18,067798	Stockholm	1	Y	Offseason	II (orange)	6-8	500-600
20	M20002	16.05.2018	28.05.2018	12	62,44088	17,423142	Medelpad	562	N	Late Spring	IV (olive)	4-6	500-600
20	M20005	13.06.2018	23.06.2018	10	62,44088	17,423142	Medelpad	188	N	Midsummer	IV (olive)	4-6	500-600
20	M20011	23.07.2018	01.08.2018	9	62,44088	17,423142	Medelpad	181	N	Late Summer	IV (olive)	4-6	500-600
20	M20020	29.10.2018	15.11.2018	17	62,44088	17,423142	Medelpad	35	Y	Offseason	IV (olive)	4-6	500-600
21	M21002	15.05.2018	29.05.2018	14	63,34501	14,537859	Jämtland	551	N	Late Spring	VI (purple)	2-4	500-600
21	M21004	12.06.2018	25.06.2018	13	63,34501	14,537859	Jämtland	260	N	Midsummer	VI (purple)	2-4	500-600
21	M21007	23.07.2018	06.08.2018	14	63,34501	14,537859	Jämtland	179	N	Late Summer	VI (purple)	2-4	500-600
21	M21014	30.10.2018	12.11.2018	13	63,34501	14,537859	Jämtland	2	Y	Offseason	VI (purple)	2-4	500-600
22	M22003	17.05.2018	24.05.2018	7	62,6455	15,63441	Jämtland	563	N	Late Spring	VII (dark blue)	2-4	500-600
22	M22006	11.06.2018	22.06.2018	11	62,6455	15,63441	Jämtland	180	N	Midsummer	VII (dark blue)	2-4	500-600

22	M22012	28.07.2018	05.08.2018	8	62,6455	15,63441	Jämtland	172	N	Late Summer	VII (dark blue)	2-4	500-600
22	M22018	14.10.2018	04.11.2018	21	62,6455	15,63441	Jämtland	94	Y	Offseason	VII (dark blue)	2-4	500-600
23	M23001	16.05.2018	23.05.2018	7	63,8201	20,316182	Västerbotten	301	Y	Late Spring	V (green)	4-6	600-700
23	M23006	20.06.2018	26.06.2018	6	63,8201	20,316182	Västerbotten	179	N	Midsummer	V (green)	4-6	600-700
23	M23011	25.07.2018	01.08.2018	7	63,8201	20,316182	Västerbotten	186	N	Late Summer	V (green)	4-6	600-700
23	M23014	26.09.2018	07.11.2018	42	63,8201	20,316182	Västerbotten	55	Y	Offseason	V (green)	4-6	600-700
24	M24002	20.05.2018	28.05.2018	8	63,79557	20,897138	Västerbotten	288	Y	Late Spring	V (green)	4-6	500-600
24	M24006	18.06.2018	24.06.2018	6	63,79557	20,897138	Västerbotten	189	N	Midsummer	V (green)	4-6	500-600
24	M24013	26.07.2018	05.08.2018	10	63,79557	20,897138	Västerbotten	170	N	Late Summer	V (green)	4-6	500-600
24	M24018	11.10.2018	14.11.2018	34	63,79557	20,897138	Västerbotten	37	Y	Offseason	V (green)	4-6	500-600
25	M25001	01.06.2018	24.06.2018	23	66,42998	20,624348	Norrbotten	542	N	Midsummer	VI (purple)	0-2	600-700
25	M25004	22.07.2018	07.08.2018	16	66,42998	20,624348	Norrbotten	172	N	Late Summer	VI (purple)	0-2	600-700
25	M25007	25.10.2018	15.11.2018	21	66,42998	20,624349	Norrbotten	no phorids	n/a	Offseason	VI (purple)	0-2	600-700
26	M26001	02.06.2018	24.06.2018	22	66,37515	20,713693	Norrbotten	545	N	Midsummer	VI (purple)	0-2	600-700
26	M26004	22.07.2018	07.08.2018	16	66,37515	20,713693	Norrbotten	183	N	Late Summer	VI (purple)	0-2	600-700
26	M26007	25.10.2018	15.11.2018	21	66,37515	20,713694	Norrbotten	no phorids	n/a	Offseason	VI (purple)	0-2	600-700
28	M28001	05.06.2018	28.06.2018	23	68,11062	23,327608	Norrbotten	545	N	Midsummer	VIII (light blue)	-2-0	500-600
28	M28007	25.07.2018	01.08.2018	7	68,11062	23,327608	Norrbotten	183	N	Late Summer	VIII (light blue)	-2-0	500-600
28	M28010	29.09.2018	25.05.2019	238	68,11062	23,327608	Norrbotten	184	N	Offseason	VIII (light blue)	-2-0	500-600
29	M29001	05.06.2018	28.06.2018	23	68,07975	23,243206	Norrbotten	500	N	Midsummer	VIII (light blue)	-2-0	500-600
29	M29007	25.07.2018	03.08.2018	9	68,07975	23,243206	Norrbotten	185	N	Late Summer	VIII (light blue)	-2-0	500-600
29	M29009	18.08.2018	07.07.2019	323	68,07975	23,243206	Norrbotten	186	N	Offseason	VIII (light blue)	-2-0	500-600
30	M30001	05.06.2018	28.06.2018	23	68,11143	23,330286	Norrbotten	174	N	Midsummer	VIII (light blue)	-2-0	500-600
30	M30007	25.07.2018	01.08.2018	7	68,11143	23,330286	Norrbotten	188	N	Late Summer	VIII (light blue)	-2-0	500-600
30	M30010	29.09.2018	02.07.2019	276	68,11143	23,330286	Norrbotten	187	N	Offseason	VIII (light blue)	-2-0	500-600
31	M31001	03.06.2018	03.07.2018	30	68,35478	18,822478	Norrbotten	503	N	Midsummer	Alpine (white)	0-2	300-400
31	M31005	29.07.2018	07.08.2018	9	68,35478	18,822478	Norrbotten	187	N	Late Summer	Alpine (white)	0-2	300-400
31	M31013	04.10.2018	27.05.2019	235	68,35478	18,822478	Norrbotten	186	N	Offseason	Alpine (white)	0-2	300-400
32	M32001	03.06.2018	03.07.2018	30	68,35515	18,826526	Norrbotten	531	N	Midsummer	Alpine (white)	0-2	300-400
32	M32005	29.07.2018	07.08.2018	9	68,35515	18,826526	Norrbotten	178	N	Late Summer	Alpine (white)	0-2	300-400
32	M32013	04.10.2018	27.05.2019	235	68,35515	18,826526	Norrbotten	189	N	Offseason	Alpine (white)	0-2	300-400
33	M33001	03.06.2018	03.07.2018	30	68,36345	18,763011	Norrbotten	523	N	Midsummer	Alpine (white)	0-2	300-400
33	M33005	29.07.2018	07.08.2018	9	68,36345	18,763011	Norrbotten	183	N	Late Summer	Alpine (white)	0-2	300-400
33	M33013	04.10.2018	27.05.2019	235	68,36345	18,763011	Norrbotten	187	N	Offseason	Alpine (white)	0-2	300-400
34	M34001	03.06.2018	03.07.2018	30	68,35441	18,827631	Norrbotten	542	N	Midsummer	Alpine (white)	0-2	300-400
34	M34005	29.07.2018	07.08.2018	9	68,35441	18,827631	Norrbotten	180	N	Late Summer	Alpine (white)	0-2	300-400
34	M34013	04.10.2018	28.05.2019	236	68,35441	18,827631	Norrbotten	187	N	Offseason	Alpine (white)	0-2	300-400
35	M35001	03.06.2018	03.07.2018	30	68,36101	18,736851	Norrbotten	554	N	Midsummer	Alpine (white)	0-2	300-400
35	M35005	29.07.2018	07.08.2018	9	68,36101	18,736851	Norrbotten	176	N	Late Summer	Alpine (white)	0-2	300-400
35	M35013	04.10.2018	16.06.2019	255	68,36101	18,736851	Norrbotten	188	N	Offseason	Alpine (white)	0-2	300-400
38	M38001	01.06.2018	15.06.2018	14	65,95729	16,205235	Västerbotten	525	N	Late Spring	VIII (light blue)	0-2	600-700
38	M38002	15.06.2018	02.07.2018	17	65,95729	16,205235	Västerbotten	142	N	Midsummer	VIII (light blue)	0-2	600-700
38	M38004	22.07.2018	17.08.2018	26	65,95729	16,205235	Västerbotten	183	N	Late Summer	VIII (light blue)	0-2	600-700
38	M38008	19.10.2018	09.11.2018	21	65,95729	16,205235	Västerbotten	6	Y	Offseason	VIII (light blue)	0-2	600-700
40	M40013	10.06.2018	25.06.2018	15	55,52346	12,910872	Skåne	166	N	Midsummer	I (red)	8-10	500-600
40	M40014	14.05.2018	28.05.2018	14	55,52346	12,910872	Skåne	218	N	Late Spring	I (red)	8-10	500-600
40	M40017	19.07.2018	07.08.2018	19	55,52346	12,910872	Skåne	155	N	Late Summer	I (red)	8-10	500-600
40	M40023	12.10.2018	10.11.2018	29	55,52347	12,910873	Skåne	5	Y	Offseason	I (red)	8-10	500-600
41	M41006	14.05.2018	22.05.2018	8	57,68907	11,956801	Västergötland	443	Y	Late Spring	II (orange)	8-10	900-1000
41	M41011	19.06.2018	26.06.2018	7	57,68907	11,956801	Västergötland	156	N	Midsummer	II (orange)	8-10	900-1000
41	M41016	26.07.2018	02.08.2018	7	57,68907	11,956801	Västergötland	180	N	Late Summer	II (orange)	8-10	900-1000
41	M41022	12.10.2018	05.11.2018	24	57,68907	11,956801	Västergötland	171	N	Offseason	II (orange)	8-10	900-1000
42	M42003	12.10.2018	10.11.2018	29	55,52382	12,911576	Skåne	15	Y	Offseason	I (red)	8-10	500-600
42	M42004	19.07.2018	07.08.2018	19	55,52382	12,911575	Skåne	170	N	Late Summer	I (red)	8-10	500-600
42	M42025	14.05.2018	28.05.2018	14	55,52382	12,911575	Skåne	636	N	Late Spring	I (red)	8-10	500-600
42	M42030	10.06.2018	25.06.2018	15	55,52382	12,911575	Skåne	174	N	Midsummer	I (red)	8-10	500-600
43	M43001	05.06.2018	28.06.2018	23	68,11132	23,331281	Norrbotten	557	N	Midsummer	VIII (light blue)	-2-0	500-600
43	M43007	25.07.2018	01.08.2018	7	68,11132	23,331281	Norrbotten	185	N	Late Summer	VIII (light blue)	-2-0	500-600
43	M43010	29.09.2018	20.07.2019	294	68,11132	23,331281	Norrbotten	187	N	Offseason	VIII (light blue)	-2-0	500-600
44	M44002	15.05.2018	29.05.2018	14	63,34467	14,536979	Jämtland	524	N	Late Spring	VI (purple)	2-4	500-600
44	M44004	12.06.2018	25.06.2018	13	63,34467	14,536979	Jämtland	169	N	Midsummer	VI (purple)	2-4	500-600
44	M44007	23.07.2018	06.08.2018	14	63,34467	14,536979	Jämtland	184	N	Late Summer	VI (purple)	2-4	500-600
44	M44014	30.10.2018	12.11.2018	13	63,34467	14,536979	Jämtland	1	Y	Offseason	VI (purple)	2-4	500-600
45	M45002	20.05.2018	28.05.2018	8	63,79621	20,900016	Västerbotten	63	Y	Late Spring	V (green)	4-6	500-600
45	M45006	18.06.2018	24.06.2018	6	63,79621	20,900016	Västerbotten	64	Y	Midsummer	V (green)	4-6	500-600
45	M45013	26.07.2018	05.08.2018	10	63,79621	20,900016	Västerbotten	179	N	Late Summer	V (green)	4-6	500-600
45	M45018	11.10.2018	14.11.2018	34	63,79621	20,900016	Västerbotten	24	Y	Offseason	V (green)	4-6	500-600
46	M46001	04.07.2018	12.07.2018	8	56,61953	16,498101	Kalmar	932	N	Midsummer	I (red)	6-8	500-600
46	M46004	31.07.2018	30.08.2018	30	56,61953	16,498101	Kalmar	178	N	Late Summer	I (red)	6-8	500-600
46	M46006	15.10.2018	01.01.2019	78	56,61953	16,498101	Kalmar	48	Y	Offseason	I (red)	6-8	500-600

Supplementary Table S2. Distinctness of scuttle fly communities across zones, as based on different clustering thresholds for species delimitation.

*Species turnover across zones, assessed using ANOSIM. Values above the grey diagonal line are R-statistics, whereas values below the diagonal line are P-values (Significance codes: 0 '***' 0.001 '**' 0.01 '*' 0.05 '.' 0.1 ' ' 1) for haplotypes and mOTUs delimited at thresholds of 0.5%, 1.7%, 3%, 4%, and 5%.*

ANOSIM(Haplo) R statistic above diagonal Significance level below diagonal
Sample statistic: 0.539 Significance of sample statistic: 0.001 Number of permutations: 999

	Zone 1	Zone 2	Zone 3	Zone 4	Zone 5	Zone 6	Zone 7	Zone 8	Alpine zone
Zone 1		0,309	0,189	0,481	0,662	0,668	0,739	0,783	0,899
Zone 2	0,002		0,233	0,312	0,661	0,588	0,705	0,567	0,626
Zone 3	0,132	0,062		0,593	0,571	0,546	0,778	0,454	0,289
Zone 4	0,006	0,018	0,100		0,369	0,142	0,185	0,331	0,252
Zone 5	0,001	0,001	0,017	0,025		0,227	0,321	0,228	0,238
Zone 6	0,001	0,001	0,010	0,171	0,029		0,193	0,145	0,256
Zone 7	0,002	0,002	0,100	0,300	0,033	0,112		0,248	0,214
Zone 8	0,001	0,001	0,012	0,042	0,030	0,039	0,074		0,156
Alpine zone	0,001	0,001	0,039	0,049	0,023	0,007	0,072	0,023	

ANOSIM(0.5%) R statistic above diagonal Significance level below diagonal
Sample statistic: 0.539 Significance of sample statistic: 0.001 Number of permutations: 999

	Zone 1	Zone 2	Zone 3	Zone 4	Zone 5	Zone 6	Zone 7	Zone 8	Alpine zone
Zone 1		0,334	0,064	0,488	0,692	0,695	0,774	0,796	0,870
Zone 2	0,001		0,196	0,344	0,654	0,593	0,751	0,592	0,544
Zone 3	0,323	0,110		0,444	0,536	0,538	0,630	0,480	0,290
Zone 4	0,004	0,024	0,200		0,294	0,143	0,111	0,346	0,181
Zone 5	0,001	0,001	0,017	0,058		0,212	0,278	0,243	0,236
Zone 6	0,001	0,001	0,010	0,161	0,033		0,208	0,151	0,196
Zone 7	0,001	0,002	0,100	0,500	0,075	0,105		0,272	0,145
Zone 8	0,001	0,001	0,007	0,032	0,014	0,033	0,049		0,101
Alpine zone	0,001	0,001	0,036	0,097	0,017	0,021	0,118	0,052	

ANOSIM(1.7%) R statistic above diagonal Significance level below diagonal
Sample statistic: 0.512 Significance of sample statistic: 0.001 Number of permutations: 999

	Zone 1	Zone 2	Zone 3	Zone 4	Zone 5	Zone 6	Zone 7	Zone 8	Alpine zone
Zone 1		0,326	0,074	0,472	0,643	0,640	0,716	0,765	0,877
Zone 2	0,002		0,217	0,297	0,675	0,517	0,757	0,602	0,601
Zone 3	0,291	0,099		0,481	0,579	0,486	0,556	0,492	0,461
Zone 4	0,016	0,031	0,200		0,298	0,049	0,111	0,287	0,205
Zone 5	0,001	0,001	0,017	0,067		0,156	0,286	0,199	0,226
Zone 6	0,001	0,001	0,010	0,315	0,076		0,149	0,153	0,223
Zone 7	0,001	0,002	0,100	0,600	0,058	0,154		0,223	0,173
Zone 8	0,001	0,001	0,006	0,050	0,032	0,041	0,071		0,095
Alpine zone	0,001	0,001	0,013	0,086	0,024	0,020	0,105	0,063	

ANOSIM(3%) R statistic above diagonal Significance level below diagonal
Sample statistic: 0.466 Significance of sample statistic: 0.001 Number of permutations: 999

	Zone 1	Zone 2	Zone 3	Zone 4	Zone 5	Zone 6	Zone 7	Zone 8	Alpine zone
Zone 1		0,279	0,085	0,415	0,522	0,579	0,726	0,612	0,670
Zone 2	0,002		0,273	0,244	0,575	0,526	0,782	0,580	0,749
Zone 3	0,289	0,062		0,481	0,516	0,431	0,519	0,556	0,931
Zone 4	0,060	0,035	0,200		0,286	0,017	0,000	0,311	0,867
Zone 5	0,001	0,001	0,025	0,042		0,111	0,385	0,281	0,766
Zone 6	0,001	0,001	0,010	0,399	0,137		0,108	0,195	0,574
Zone 7	0,001	0,002	0,100	0,600	0,033	0,199		0,351	0,793
Zone 8	0,001	0,001	0,001	0,025	0,015	0,013	0,017		0,248
Alpine zone	0,001	0,001	0,001	0,001	0,001	0,001	0,001	0,002	

ANOSIM(4%)	R statistic above diagonal			Significance level below diagonal					
Sample statistic: 0.399		Significance of sample statistic: 0.001				Number of permutations: 999			
	Zone 1	Zone 2	Zone 3	Zone 4	Zone 5	Zone 6	Zone 7	Zone 8	Alpine zone
Zone 1		0,267	0,101	0,419	0,487	0,548	0,686	0,523	0,540
Zone 2	0,002		0,260	0,194	0,391	0,452	0,713	0,510	0,634
Zone 3	0,291	0,099		0,556	0,496	0,506	0,630	0,661	0,972
Zone 4	0,016	0,031	0,200		0,254	0,014	0,111	0,242	0,843
Zone 5	0,001	0,001	0,017	0,067		0,082	0,337	0,242	0,693
Zone 6	0,001	0,001	0,010	0,315	0,076		0,129	0,203	0,620
Zone 7	0,001	0,002	0,100	0,600	0,058	0,154		0,299	0,809
Zone 8	0,001	0,001	0,006	0,050	0,032	0,041	0,071		0,271
Alpine zone	0,001	0,001	0,013	0,086	0,024	0,020	0,105	0,063	

ANOSIM(5%)	R statistic above diagonal			Significance level below diagonal					
Sample statistic: 0.270		Significance of sample statistic: 0.001				Number of permutations: 999			
	Zone 1	Zone 2	Zone 3	Zone 4	Zone 5	Zone 6	Zone 7	Zone 8	Alpine zone
Zone 1		0,181	0,228	0,366	0,468	0,408	0,447	0,408	0,319
Zone 2	0,006		0,425	0,267	0,332	0,317	0,513	0,351	0,444
Zone 3	0,700	0,022		0,593	0,540	0,601	0,519	0,740	0,955
Zone 4	0,002	0,044	0,100		0,119	0,053	-0,037	0,238	0,799
Zone 5	0,001	0,003	0,008	0,242		0,159	0,234	0,285	0,676
Zone 6	0,001	0,003	0,003	0,301	0,063		0,154	0,212	0,584
Zone 7	0,001	0,002	0,100	0,600	0,108	0,143		0,373	0,785
Zone 8	0,001	0,001	0,001	0,070	0,006	0,100	0,009		0,256
Alpine zone	0,001	0,001	0,001	0,002	0,001	0,001	0,002	0,002	

Supplementary Table S3. Similarity of scuttle fly communities across zones, as based on different clustering thresholds for species delimitation.

Shown are results of SIMPER analyses of species turnover across zones, for haplotypes and mOTUs delimited at thresholds of 0.5%, 1.7%, 3%, 4%, and 5%.

SIMPER(Haplo)										
		Between Zone Similarity (%)								
	Within Zone Similarity (%)	Zone 1	Zone 2	Zone 3	Zone 4	Zone 5	Zone 6	Zone 7	Zone 8	Alpine zone
Zone 1	18,32									
Zone 2	18,75	14,03								
Zone 3	22,81	15,32	15,15							
Zone 4	23,29	11,88	14,06	15,83						
Zone 5	21,5	9,75	9,78	12,94	16,28					
Zone 6	22,19	9,51	11,46	12,85	18,89	17,76				
Zone 7	18,78	8,45	8,67	11,87	19,48	16,20	18,38			
Zone 8	18,67	6,94	8,50	9,08	11,30	14,15	16,50	13,03		
Alpine zone	17,75	3,27	3,74	4,95	5,98	8,15	10,20	8,14	13,24	

SIMPER(0.5%)										
		Between Zone Similarity (%)								
	Within Zone Similarity (%)	Zone 1	Zone 2	Zone 3	Zone 4	Zone 5	Zone 6	Zone 7	Zone 8	Alpine zone
Zone 1	24,3									
Zone 2	24,41	18,83								
Zone 3	29,45	22,63	20,8							
Zone 4	28,75	16,19	18,56	21,81						
Zone 5	26,03	13,16	13,42	16,87	21,41					
Zone 6	28,53	12,9	15,26	17,54	24,61	22,84				
Zone 7	22,93	11,29	11,18	16,2	24,62	20,46	23,62			
Zone 8	24,59	9,75	11,56	12,33	15,48	18,48	21,7	17,29		
Alpine zone	22,7	5,78	6,97	7,23	10,01	11,31	15,51	12,26	18,98	

SIMPER(1.7%)

	Within Zone Similarity (%)	Zone 1	Zone 2	Zone 3	Zone 4	Zone 5	Zone 6	Zone 7	Zone 8	Alpine zone
		Between Zone Similarity (%)								
Zone 1	26,58									
Zone 2	26,76	21,17								
Zone 3	31,00	24,78	23,12							
Zone 4	31,12	18,54	22,09	23,16						
Zone 5	30,25	15,65	16,83	18,99	25,22					
Zone 6	30,51	15,45	19,30	19,76	28,20	26,54				
Zone 7	24,63	13,79	15,01	17,82	27,61	23,90	26,57			
Zone 8	27,83	12,03	14,59	14,94	20,01	22,94	24,43	21,48		
Alpine zone	27,85	7,82	10,58	9,13	14,20	16,96	18,58	16,21	23,34	

SIMPER(3%)

	Within Zone Similarity (%)	Zone 1	Zone 2	Zone 3	Zone 4	Zone 5	Zone 6	Zone 7	Zone 8	Alpine zone
		Between Zone Similarity (%)								
Zone 1	28,65									
Zone 2	29,34	24,24								
Zone 3	33,58	26,57	24,54							
Zone 4	32,51	22,1	25,27	25,83						
Zone 5	32,95	21,05	21,4	23,21	28,28					
Zone 6	21,12	19,95	21,83	22,79	30,33	29,28				
Zone 7	26,49	17,75	17,75	21,25	30,69	25,18	28,8			
Zone 8	32,74	19,43	20,89	20,63	25,54	26,51	27,94	24,72		
Alpine zone	42,98	19,01	22	18,42	22,23	23,19	25,82	22,07	31,1	

SIMPER(4%)

	Within Zone Similarity (%)	Zone 1	Zone 2	Zone 3	Zone 4	Zone 5	Zone 6	Zone 7	Zone 8	Alpine zone
		Between Zone Similarity (%)								
Zone 1	31,14									
Zone 2	31,54	26,99								
Zone 3	36,58	28,86	26,95							
Zone 4	35,64	24,58	27,98	26,58						
Zone 5	34,36	24,07	26,03	24,9	30,76					
Zone 6	34,78	22,83	25,2	24,48	33,88	32,12				
Zone 7	29,92	20,92	21,8	23,14	33,29	27,78	31,91			
Zone 8	36,37	23,26	25,28	22,51	31,01	30,97	31,34	29,95		
Alpine zone	50,63	23,93	27,77	20,88	29,72	30,16	30,24	28,48	36,41	

SIMPER(5%)

	Within Zone Similarity (%)	Zone 1	Zone 2	Zone 3	Zone 4	Zone 5	Zone 6	Zone 7	Zone 8	Alpine zone
		Between Zone Similarity (%)								
Zone 1	35,24									
Zone 2	39,81	32,44								
Zone 3	38,77	30,97	30,71							
Zone 4	39,31	28,82	34,37	28,22						
Zone 5	37,94	27,45	33,14	27,08	36					
Zone 6	41,08	28,29	33,91	28,38	39,25	36,16				
Zone 7	35,39	27,73	31,71	28,12	39,45	33,31	37,9			
Zone 8	42,79	28,78	34,95	26,97	37,83	36,36	37,91	35,46		
Alpine zone	62,06	31,66	40,94	28,65	39,99	38,97	39,78	38,92	45,42	

Supplementary Table S4. Distinctness of scuttle fly communities across time periods.

Shown are results of ANOSIM analyses of species turnover across time-periods for all zones (top) and northern (IV-alpine) zones only (bottom).

ANOSIM	R=0.269, P=0.001			
	Late Spring	Midsummer	Late Summer	Offseason
Late Spring		0,091	0,350	0,390
Midsummer	0,018		0,262	0,233
Late Summer	0,001	0,001		0,339
Offseason	0,001	0,002	0,001	

ANOSIM	R=0.588, P=0.001)			
	Late Spring	Midsummer	Late Summer	Offseason
Late Spring		0.104	0,882	0,728
Midsummer	0,125		0,712	0,383
Late Summer	0,001	0,001		0,826
Offseason	0,001	0,003	0,001	

Supplementary Table S5. Similarity of scuttle fly communities across time periods.

Shown are results of SIMPER analyses of species turnover across time-periods for all zones (top) and northern (IV-alpine) zones only (bottom).

SIMPER	Within Season Similarity (%)	Between Season Similarity (%)			
		Late Spring	Midsummer	Late Summer	Offseason
Late Spring	26,52				
Midsummer	23,57	22,12			
Late Summer	20,61	14,45	16,40		
Offseason	24,23	16,58	18,59	13,12	

SIMPER	Within Season Similarity (%)	Between Season Similarity (%)			
		Late Spring	Midsummer	Late Summer	Offseason
Late Spring	42,93				
Midsummer	32,81	31,55			
Late Summer	29,30	10,62	16,25		
Offseason	43,58	29,03	26,45	13,87	

Supplementary Table S6. Mantel tests relating pairwise community dissimilarity to pairwise differences in space, time or both.

To examine the effect of the species delimitation criteria used, we computed pairwise Jaccard community dissimilarity values using both individual haplotypes (dataset "Haplotypes") and species defined by 1.7% sequence divergence (dataset "Species"). Total dissimilarity (Total Betadiversity) was resolved into its Turnover and Nestedness components. Each component was then related to the spatial difference (i.e., the geographical distance between sites), the temporal difference (i.e., temporal distance in weeks between samples) and the spatio-temporal differences (i.e., geographical distance and temporal distance, including their joint effect) between sample pairs. Columns r *denote Pearson moment product correlations, whereas* p*-values are based on Mantel tests.*

Dataset	Distances	Turnover		Nestedness		Total Betadiversity	
		r	*p*	*r*	*p*	*r*	*p*
Species	Spatial	**0.75**	**0.001**	-0.33	1	**0.65**	**0.001**
	Temporal	-0.03	0.5	0.53	0.21	0.58	0.125
	SpatioTemporal – Late spring	**0.57**	**0.001**	-0.19	0.997	**0.64**	**0.001**
	SpatioTemporal – Midsummer	**0.64**	**0.001**	-0.31	1	**0.61**	**0.001**
	SpatioTemporal – Late Summer	**0.74**	**0.001**	-0.41	1	**0.73**	**0.001**
	SpatioTemporal – Offseason	**0.25**	**0.001**	-0.25	1	**0.43**	**0.001**
Haplotypes	Spatial	**0.79**	**0.001**	-0.37	1	**0.69**	**0.001**
	Temporal	-0.73	0.92	0.93	0.083	**0.69**	**0.041**
	SpatioTemporal – Late spring	**0.64**	**0.001**	-0.34	1	**0.60**	**0.001**
	SpatioTemporal – Midsummer	**0.63**	**0.001**	-0.24	1	**0.62**	**0.001**
	SpatioTemporal – Late Summer	**0.72**	**0.001**	-0.43	1	**0.71**	**0.001**
	SpatioTemporal – Offseason	**0.14**	**0.001**	-0.17	1	**0.42**	**0.001**
